# Identification of QTL on Chromosome 18 Associated with Non-Coagulating Milk in Swedish Red Cows

**DOI:** 10.3389/fgene.2016.00057

**Published:** 2016-04-15

**Authors:** Sandrine I. Duchemin, Maria Glantz, Dirk-Jan de Koning, Marie Paulsson, Willem F. Fikse

**Affiliations:** ^1^Department of Animal Breeding and Genetics, Swedish University of Agricultural SciencesUppsala, Sweden; ^2^Animal Breeding and Genomics Centre, Wageningen UniversityWageningen, Netherlands; ^3^Department of Food Technology, Engineering and Nutrition, Lund UniversityLund, Sweden

**Keywords:** non-coagulating milk, sequences, dairy, cheese production, haplotypes, *VPS35*

## Abstract

Non-coagulating (**NC**) milk, defined as milk not coagulating within 40 min after rennet-addition, can have a negative influence on cheese production. Its prevalence is estimated at 18% in the Swedish Red (**SR**) cow population. Our study aimed at identifying genomic regions and causal variants associated with NC milk in SR cows, by doing a GWAS using 777k SNP genotypes and using imputed sequences to fine map the most promising genomic region. Phenotypes were available from 382 SR cows belonging to 21 herds in the south of Sweden, from which individual morning milk was sampled. NC milk was treated as a binary trait, receiving a score of one in case of non-coagulation within 40 min. For all 382 SR cows, 777k SNP genotypes were available as well as the combined genotypes of the genetic variants of αs1-β-κ-caseins. In addition, whole–genome sequences from the 1000 Bull Genome Consortium (Run 3) were available for 429 animals of 15 different breeds. From these sequences, 33 sequences belonged to SR and Finish Ayrshire bulls with a large impact in the SR cow population. Single-marker analyses were run in ASReml using an animal model. After fitting the casein loci, 14 associations at −Log_10_(*P*-value) > 6 identified a promising region located on BTA18. We imputed sequences to the 382 genotyped SR cows using Beagle 4 for half of BTA18, and ran a region-wide association study with imputed sequences. In a seven mega base-pairs region on BTA18, our strongest association with NC milk explained almost 34% of the genetic variation in NC milk. Since it is possible that multiple QTL are in strong LD in this region, 59 haplotypes were built, genetically differentiated by means of a phylogenetic tree, and tested in phenotype-genotype association studies. Haplotype analyses support the existence of one QTL underlying NC milk in SR cows. A candidate gene of interest is the *VPS35* gene, for which one of our strongest association is an intron SNP in this gene. The *VPS35* gene belongs to the mammary gene sets of pre-parturient and of lactating cows.

## Introduction

Non- or poor-coagulating milk is an undesirable characteristic of milk with a negative influence on cheese production. Non-coagulating (**NC**) milk is prevalent among several dairy cattle breeds, such as Swedish Red (**SR**), Finnish Ayrshire (**FAY**), Holstein-Friesian (**HF**) and Italian Brown Swiss, to name a few (e.g., Cecchinato et al., 2011; Frederiksen et al., 2011; Gustavsson et al., 2014a). The prevalence of NC milk varies among these breeds ranging from 4% in Italian Brown Swiss (Cecchinato et al., 2009) up to 13% in FAY (Ikonen et al., 2004). A recent study has estimated the prevalence of NC milk, defined as milk not coagulating within 40 min after rennet-addition, at 18% in the SR cow population (Gustavsson et al., 2014a). Targeted research on NC milk can help geneticists develop breeding programs to modify milk composition and technological properties of milk and thus reduce the prevalence of NC milk.

Bittante et al. (2012) suggested that effects of herd have little influence on milk coagulation properties (**MCP**) including NC milk, although several factors can influence the composition of bovine milk (e.g., breed, a cow's diet, age of a cow, and the stage of lactation; Chilliard et al., 2001). In addition, MCP seem to be influenced by many factors, such as SCC (e.g., Ikonen et al., 2004; Cassandro et al., 2008), titratable acidity (e.g., Penasa et al., 2010), casein composition (Okigbo et al., 1985b), pH (Nájera et al., 2003), stage of lactation (Okigbo et al., 1985a; Ostersen et al., 1997), and breed (e.g., Auldist et al., 2004; De Marchi et al., 2007; Bittante et al., 2012), among many other factors. Heritability estimates for MCP and NC milk range from 0.26 in FAY (Ikonen et al., 2004) to 0.45 in SR cows (Gustavsson et al., 2014a). These heritability estimates suggest that breeding could effectively reduce the prevalence of NC milk. In Sweden, the breeding program includes production traits to guarantee the increase in both protein and fat contents (Nordic Cattle Genetic Evaluation, 2013). The negative genetic correlations between NC milk and protein content estimated by Gustavsson et al. (2014a) suggest that breeding for higher protein content in the SR cows can lead to an increase in the prevalence of NC milk. In Sweden, 41% of SR cows produce milk for the dairy industry, and more than 30% of total milk production is used for cheese production (LRF Dairy Sweden, 2015). Since, total milk production is about 3 million tons per year (LRF Dairy Sweden, 2015) and the market price of milk produced is about 0.28 euros per kg, the problem of NC milk affects milk with a value of ~63 million euros per year. Frederiksen et al. (2011) has estimated in 25% the proportion of NC milk in a batch of well-coagulating milk that is sufficient to deteriorate the MCP of well-coagulating milk. Van Hooydonk et al. (1986) showed that the addition of calcium would restore coagulation of NC milk but not to the level of well-coagulating milk according to Hallén et al. (2010). Furthermore, addition of calcium above 0.04% have been reported to produce a bitter flavor (Schwarz and Mumm, 1948) which could be detrimental to cheese production. Therefore, it is important to the Swedish industry to reduce the frequency of NC milk.

It is well-established that MCP, including NC milk, are strongly influenced by variable proportions, and genetic variants of milk protein fractions [especially of κ-casein (**CN**); (Bittante et al., 2012)]. In poor- and non-coagulating milk samples of Danish Jerseys and HF cows, Jensen et al. (2012) showed that BB-A^2^A^2^-AA was the predominant combined genotype of α_*S*1_-, β-, and κ-CN associated with NC milk. Hallén et al. (2007) and Gustavsson et al. (2014b) showed that some of these genotypes (especially β-, and κ-CN genotypes A^2^A^2^-AA) segregate in SR cows. Besides these genetic variants of milk protein fractions in the cattle genome, other undiscovered genes might play a role in the prevalence of NC milk. These genes can be identified by genome-wide association studies (**GWAS**) using high-density genotyping techniques.

High-density genotyping techniques, such as whole-genome sequences (**WGS**), can help GWAS increase the power and the precision of quantitative trait loci (**QTL**) mapping. WGS are expected to contain most of the polymorphisms causing the genetic differences between individuals (Meuwissen and Goddard, 2010). When an entire population is sequenced, WGS are independent of linkage disequilibrium (**LD**) between polymorphisms and the causal variant (Druet et al., 2014) in comparison with a lower panel of markers. However, sequencing an entire population can be expensive, and a cost-effective strategy consists of sequencing key ancestors of a population, and imputing to sequence level the rest of this population (Druet et al., 2014). To demonstrate this approach, Daetwyler et al. (2014) imputed dairy cattle populations that were genotyped with 777k SNP (**BovineHD**) to sequence level using WGS from the 1000 Bull Genome Consortium. Their study targeted some known genomic regions where QTL affecting milk production and curly coat had previously been identified, and they successfully identified the causal variants underlying these QTL. Therefore, GWAS using imputed sequences could assist in the identification of causal variants.

A recent GWAS in SR cows used BovineHD as genotypes and MCP as phenotypes (Gregersen et al., 2015). However, their GWAS did not include NC milk in the analyses. The aim of our study was to identify genomic regions and causal variants associated with NC milk in SR cows. For this purpose, firstly we ran a GWAS using BovineHD genotypes to identify the most promising genomic region associated with NC milk, and secondly we fine-mapped this genomic region using imputed sequences.

## Materials and methods

### Animals and phenotypes

Morning milk samples were retrieved from 382 SR cows belonging to 21 herds in the southern part of Sweden. Cows were kept indoors, were fed according to standard practices, and were milked 2 or 3 times a day. Cows were daughters of 160 sires, and were chosen to be as genetically unrelated as possible. Cows were multiparous, ranging from 1 through 3 parturitions, and were in different lactations stages, ranging from 2.5 through 61 weeks in lactation.

Milk samples were collected in two distinct periods: April through May 2010, and September 2010 through April 2011. Directly after collection, milk samples were cooled and transported to Lund University (Lund, Sweden), where samples were defatted by centrifugation (at 2000 × g for 30 min) to reduce the number of factors influencing coagulation properties. Fresh skimmed milk samples were preserved by adding bronopol (Sigma-Aldrich, Schnelldorf, Germany) solution of 17% wt/vol (2 μL/mL), as described in Hallén et al. (2007). For rheological measurements, these milk samples were stored at +4°C, but no longer than 3 days. Skimmed milk samples were heated to 32°C for 30 min, after which chymosin (0.44 mL/L Chy-Max Plus, 205 international milk clotting units (IMCU)/mL, Chr. Hansen A/S Hørsholm, Denmark) was added, and the resulting solution was gently stirred. The addition of the chymosin represented time zero. Measurements, such as rennet gel strength, rennet coagulation time, and yield stress of rennet-induced gels, were done and described by Gustavsson et al. (2014a). Some samples were unable to coagulate within 40 min after rennet-addition, and were defined as **NC** milk samples. When observed, NC milk was scored as one, while coagulating milk was scored as zero. Of the 382 cows that had available phenotypes on coagulation properties, 18% of these cows had NC milk.

### Genotypes

A blood sample of each of the 382 SR cows was collected for genotyping purposes. These cows were genotyped for 777,963 SNP using the Illumina BovineHD BeadChip (Illumina Inc., San Diego, CA). Quality controls of the data were performed using the R-package GenAbel (Aulchenko et al., 2007), and consisted of a minimum of 95% of non-missing SNP per called genotypes (call rate) and minor allele frequency (MAF) of a minimum of 1% for a called SNP. All SNP without a map position on the UMD 3.1 genome assembly (Zimin et al., 2009) as well as SNP on the sex chromosome were discarded. After these edits, a total of 624,302 SNP were available for further analyses.

In addition, blood samples were used to extract DNA to genotype all cows for the genetic variants of αs1-, β- and κ-caseins (**CN**) using TaqMan SNP genotyping assays (Applied Biosystems, Foster City, CA), as described in Gustavsson et al. (2014b). For these variants, the assays were distinguished among the following: αs1-CN variant A, B, C, D, and F; β-CN variants A^1^, A^2^, A^3^, B, and I; and κ-CN variants A, B, and E. In their study, combined genotypes were created by combining the genetic variants of αs1-β-κ-CN. These combined genotypes were used in the present study, and are referred to as “CNcluster.”

Whole–genome sequences were available for 428 bulls and for one cow from 15 different breeds (Run three of the 1000 Bull Genomes consortium; Daetwyler et al., 2014), representing a multi-breed reference population. Among these sequences, 33 belonged to SR and FAY bulls with a large impact in the SR cow population. All positions of the variants on sequences were aligned to the bovine genome assembly UMD3.1 (Zimin et al., 2009). Within this multi-breed reference population, positions containing both a SNP and an indel were excluded because of possible problems with alignment and sequencing.

### GWAS on BovineHD genotypes

Single-marker analyses were run in ASReml 4.0 (Beta version; Gilmour et al., 2009) using the following animal model:

(1) $$\begin{array}{l} y=\;\mu +\;herd\;+\;parity\;+\;wim\;+\;e^{-0.05*wim}+\;CNcluster\;\\ \;+\;Marker\;+\;a\;+\;e \end{array}$$

where *y* is the dependent variable; μ is the overall mean, *herd* is the covariate that describes the effect of a cow belonging to a specific herd; *parity* is the covariate that describes the effect of number of parities per cow; *wim* is the covariate that describes the effect of weeks in milk, modeled as a Wilmink curve (Wilmink, 1987); *CNcluster* is the covariate describing the effect of the combined genotypes; *Marker* is the fixed effect of a variant genotype; *a* is the random effect of animal and is assumed to be distributed as $N\;\sim \;\left(0,G\sigma_{a}^{2}\right),\;$where ***G*** is the genomic relationship matrix based on 382 animals and $\sigma_{a}^{2}$ is the additive genetic variance. We calculated the G-matrix based on the BovineHD genotypes using the software calc_grm (Calus, 2013). $\sigma_{a}^{2}\;$was estimated with a model excluding the effect of *Marker*, and was fixed in model 1. *e* is the random residual effect and is assumed to be distributed as $N\;\sim \;\left(0,I\sigma_{e}^{2}\right),\;$where ***I*** is the identity matrix and $\sigma_{e}^{2}$ is the residual variance.

The most promising genomic region with multiple signals at −Log_10_(*P*-value) ≥ 6 was imputed from the BovineHD genotypes to sequence level, and a region-wide association study (**RWAS**) was performed.

### Imputation

Imputation started by checking the BovineHD against the sequenced reference population for inconsistencies using the Conform-gt software (http://faculty.washington.edu/browning/conform-gt.html). After this check, the 382 cows were imputed from BovineHD genotypes to sequence level for half of a chromosome using Beagle version 4.0 (Browning and Browning, 2007). Beagle version 4.0 was run with the following settings: 50 for phase iterations, 50 for nthreads, and 100 for imputation iterations. To account for the nature of the different variants, we ran three imputations based on different reference populations. These imputations were named as follows: “Nordic-red-specific,” “Dairy-specific,” and “Common.” For the imputation of the “Nordic-red-specific,” the reference population used consisted of the 33 sequences belonging to SR and FAY breeds. For the imputation of the “Dairy-specific,” the reference population used consisted of the 284 sequences belonging to dairy breeds (8 breeds). For the imputation of the “Common,” the reference population used consisted of 429 sequences belonging to Nordic-red, dairy and beef breeds (15 breeds). Following this approach, each variant was imputed three times based on the three different reference populations, which resulted in different imputation accuracies (Beagle allelic-r2, **AR2**) for each variant. The genotype with the highest imputation accuracy across the three imputations was selected as the best-imputed genotype.

We calculated the average concordance between the imputed genotypes across the three different scenarios of imputation, as implemented in VCFtools version 0.1.12b (Danecek et al., 2011). Subsequently, we combined the best-imputed genotypes into one data set that was used in the RWAS.

### RWAS on imputed sequences for half a chromosome

A RWAS with imputed sequence data for the most promising region on half a chromosome was run using model 1. The imputed sequences were filtered to remove poorly imputed genotypes: only variants that were imputed with an AR2 ≥ 0.2 were included in the RWAS. Single-marker analyses were run using model 1 with one modification: the variance of the genetic effect *a* was assumed to be distributed as $N\;\sim \;\left(0,G1\sigma_{a^{*}}^{2}\right),\;$ where **G1** is the genomic relationship matrix based on 382 animals and $\sigma_{a^{*}}^{2}$ is the additive genetic variance. The **G1**-matrix was calculated using the software calc_grm (Calus, 2013). The BovineHD genotypes of half chromosome that were used in the imputation to sequence level were not included in the G1-matrix calculations. $\sigma_{a^{*}}^{2}$ was calculated before the inclusion of *Marker*, and was fixed in model 1.

The most significant association from the first RWAS (coined TagSNP1) was subsequently included as a fixed effect in model 1, and a second RWAS was run. For this second RWAS, only the variants with an AR2 ≥ 0.8 were re-analyzed and considered for further analyses, such as LD calculations and haplotype analyses.

### Haplotype analyses

The construction of haplotypes started by selecting the SNP moderately to highly correlated with TagSNP1 (LD > 0.5). LD was calculated as the squared correlation between TagSNP1 and all other SNP using PLINK version 1.9 (Purcell et al., 2007). An LD plot was produced using the R-package ggplot2 (Wickham, 2009). Next, we combined these correlated SNP into haplotypes.

For the haplotypes, we produced a phylogenetic tree using the molecular evolutionary genetics analysis (MEGA6) software, version 6.0. The MEGA6 software was developed for comparative analyses of DNA and protein sequences that aim at inferring the molecular evolutionary patterns of genes, genomes, and species over time (Kumar et al., 1994; Tamura et al., 2013). To build the phylogenetic tree, we applied the Neighbor-Joining statistical method (Saitou and Nei, 1987) with a substitution model based on the proportion of nucleotide substitutions per site between nucleotides of loaded sequences. Alignment gaps and missing information gaps were accounted for with the partial-deletion option implemented in the software, and gaps were removed when the number of ambiguous sites ≥0.95.

Subsequently, the phylogenetic tree, all phenotypes and two copies of each haplotypes per cow were supplied to TreeScan software, version 1.0 (Templeton et al., 2005). TreeScan uses the phylogenetic tree built from haplotypes in phenotype-genotype association studies. With its iterative approach, TreeScan cuts in two parts a branch of the phylogenetic tree. For part 1, all haplotypes are grouped, and treated as a single allele, say A. For part 2, all haplotypes are grouped, and treated as a single allele, say B. These alleles allow different combinations of genotypes: AA, AB, and BB. Subsequently, associations between phenotypes and genotypes (AA, AB, and BB) are statistically tested with the F-statistics of a one-way ANOVA. This iterative approach is repeated until all the branches of the phylogenetic tree have been tested. The null hypothesis considered for the inference of branches (i.e., haplotypes) is of no association between a partition and the trait of interest, which in our case was NC milk. In addition, the following settings were used in TreeScan: the number of simulations to obtain *P*-values for the ANOVA tests *p* = 5,000; the significance level α = 0.05, and the minimum number of individuals required in each observed genotypic class *c* = 2. A bipartition was considered as significantly associated to NC milk at *P* < 0.05.

### Bioinformatics and candidate genes

We used the variant effect predictor (Ve!P) online tool (at http://www.ensembl.org/info/docs/tools/vep/index.html; McLaren et al., 2010) to determine the effect of the variants (SNPs, insertions, deletions, CNVs or structural variants) on genes, transcripts, and protein sequence, as well as regulatory regions.

## Results

### GWAS on BovineHD genotypes

The GWAS on BovineHD genotypes identified many significant SNP associated with NC milk after fitting the casein loci (Supplementary Figures 1A–D). The accompanying QQ-plot indicated that a small proportion of SNP were deviating from the *x* = *y* line. This smaller proportion of SNP represented the most likely associated SNP among the thousands of non-associated SNP with NC milk. In addition, no important deviations from the *x* = *y* line were observed, suggesting no obvious signs of population stratification (Supplementary Figure 2). Fourteen of the many associations were significant at −Log_10_(*P*-value) > 6, and they are located on BTA11, BTA13, and BTA18 (Table 1). The most promising region was located on BTA18, and was distributed over a region of seven mega base-pairs (**MBP**). Because BTA18 showed the most significant association with NC milk after fitting the casein loci, we focused on this chromosome by running a RWAS using imputed sequence data.

**Table 1:** **Most significant SNP from genome-wide association study with NC milk^†^ based on BovineHD genotypes in 382 Swedish Red cows**.

Chromosome	SNP	Position	−Log_10_(*P*-value)	$\sigma_{marker}^{2}$^§^	$\sigma_{marker}^{2}/\sigma_{p}^{2}$^*^
11	rs136987882	55787730	6.29	0.01	0.07
13	rs136185829	47744740	6.15	0.01	0.07
13	rs109492822	47749851	6.15	0.01	0.07
13	rs134756836	47754335	6.15	0.01	0.07
18	rs137544086	9179722	6.19	0.01	0.07
18	rs41865365	11166809	8.77	0.01	0.09
18	rs110267892	13136171	6.65	0.01	0.07
18	rs109208214	13934856	10.18	0.02	0.11
18	rs135171892	13939170	10.18	0.02	0.11
18	rs137827420	13943440	10.18	0.02	0.11
18	rs137429187	13960525	10.18	0.02	0.11
18	rs132908573	13967910	10.18	0.02	0.11
18	rs110637786	15017982	9.35	0.01	0.10
18	rs110615481	15047675	6.54	0.01	0.08

† *NC milk as binary trait where 0 = coagulating milk and 1 = non-coagulating milk*.

§ *$\sigma_{\text{marker}}^{\text{2}}\;=$ marker's variance, computed for each marker as 2 times major allele frequency times minor allele frequency times the square of the allele substitution effect*.

* *$\sigma_{marker}^{2}∕\sigma_{p}^{2}\;=$ proportion of phenotypic variance explained by a marker*.

### Imputation for half of BTA18

Before imputation, the inconsistencies between the BovineHD genotypes and the sequence data were strand problems (i.e., 1 for Nordic-red-specific, 815 for Dairy-specific, and 927 for commons), and seven SNP from the BovineHD genotypes not present in the sequence data. All these inconsistencies were set to missing in the BovineHD data, and imputed.

After imputation, the total number of variants in the region between 0–30 MBP on BTA18 increased from 7873 SNP on the BovineHD to 562,432 variants on the sequence level, representing an increase of 71 times in the total number of variants. From the 562,432 imputed variants, 69.3% were monomorphic (MAF = 0), 24.5% were polymorphic and AR2 ≥ 0.2, and 14.3% were polymorphic and AR2 ≥ 0.8 (Table 2). After filtering out the monomorphic variants, 137,949 polymorphic variants imputed with an AR2 ≥ 0.2 were left. This is an increase of more than 17 times in the total number of variants from BovineHD genotypes (*N* = 7873 SNP) to sequence level (*N* = 137,949 sites). These 137,949 variants originated from the three scenarios as follows: 98,152 variants from the Nordic-red-specific scenario, plus 28,394 variants from the Dairy-specific scenario, plus 11,403 variants from the common scenario. In addition, the 98,152 variants from the Nordic-red-specific scenario are composed of 91,363 SNP, 6753 indels, and 36 multi-allelic variants. The 28,394 variants from the Dairy-specific scenario are composed of 27,113 SNP, 1253 indels, and 28 multi-allelic variants. The 11,403 variants from the common scenario are composed of 10,989 SNP, 401 indels, and 13 multi-allelic variants.

**Table 2:** **Distribution of the average accuracy of imputation (AR2) per ranges of minor allele frequency (MAF), and the number of markers (as counts and in percentage) for the three scenarios of imputation**.

**MAF**	**AR2^†^**	**Nordic-red specific**		**Dairy-specific**		**Common**		**Total number of variants**	
		**Average AR2**	**N1^§^**	**Average AR2**	N2^*^	**Average AR2**	**N3^¢^**	**N^¥^**	**(%)**
0	All	0.00	389,518	0.00	94	0.00	54	389,666	69.3
	≥0.2	–	0	0.31	4	0.31	0	4	0.0
	≥0.8	–	–	–	–	–	–	–	–
0–0.1	All	0.42	28,346	0.37	17,720	0.34	9,547	55,613	9.9
	≥0.2	0.72	16,772	0.62	11,266	0.59	5,861	33,899	6.0
	≥0.8	0.94	9,467	0.91	2,748	0.90	1,082	13,297	2.4
0.1–0.2	All	0.69	23,425	0.63	6,951	0.61	2,774	33,150	5.9
	≥0.2	0.76	20,922	0.70	6,136	0.67	2,462	29,520	5.2
	≥8	0.94	12,994	0.92	2,329	0.91	761	16,084	2.9
0.2–0.3	All	0.75	22,765	0.70	4,593	0.68	1,467	28,825	5.1
	≥0.2	0.80	21,235	0.75	4,075	0.73	1,291	26,601	4.7
	≥0.8	0.94	14,609	0.92	2,049	0.92	412	17,070	3.0
0.3–0.4	All	0.78	21,900	0.74	4,422	0.71	1,205	27,527	4.9
	≥0.2	0.83	20,429	0.80	3,759	0.78	991	25,179	4.5
	≥0.8	0.95	15,189	0.93	2,186	0.92	392	17,767	3.2
0.4–0.5	All	0.67	22,731	0.62	3,854	0.59	1,066	27,651	4.9
	≥0.2	0.83	18,794	0.79	3,154	0.78	798	22,746	4.0
	≥0.8	0.95	13,937	0.93	1,779	0.92	272	15,988	2.8
Total	All	0.55	508,685	0.51	37,634	0.49	16,113	562,432	100.0
	≥0.2	0.79	98,152	0.73	28,394	0.71	11,403	137,949	24.5
	≥0.8	0.94	66,196	0.92	11,091	0.91	2,919	80,206	14.3

† *where: All considers all imputed animals, ≥ 0.2 considers animals imputed with an AR2 equal and higher than 0.2, and ≥ 0.8 considers animals imputed with an AR2 equal and higher than 0.8*.

§ *N1, total number of markers for the Nordic-Red specific scenario*.

* *N2, total number of markers for the Dairy-specific scenario*.

¢ *N3, total number of markers for the Common scenario*.

¥ *N, sum of markers for all three imputation scenarios (N1 + N2 + N3)*.

The average concordance was calculated by comparing genotypes imputed in the three different scenarios, and reported sites were alleles in exact match between files. Results indicated that 97.0% of the imputed genotypes from the Nordic-red-specific scenario were concordant with the Dairy-specific scenario; 96.8% of the imputed genotypes from the Nordic-red-specific scenario were concordant with the common scenario; and 98.9% of the imputed genotypes from the Dairy-specific scenario were concordant with the common scenario.

### RWAS on imputed sequences for half of BTA18

**Figure 1:**
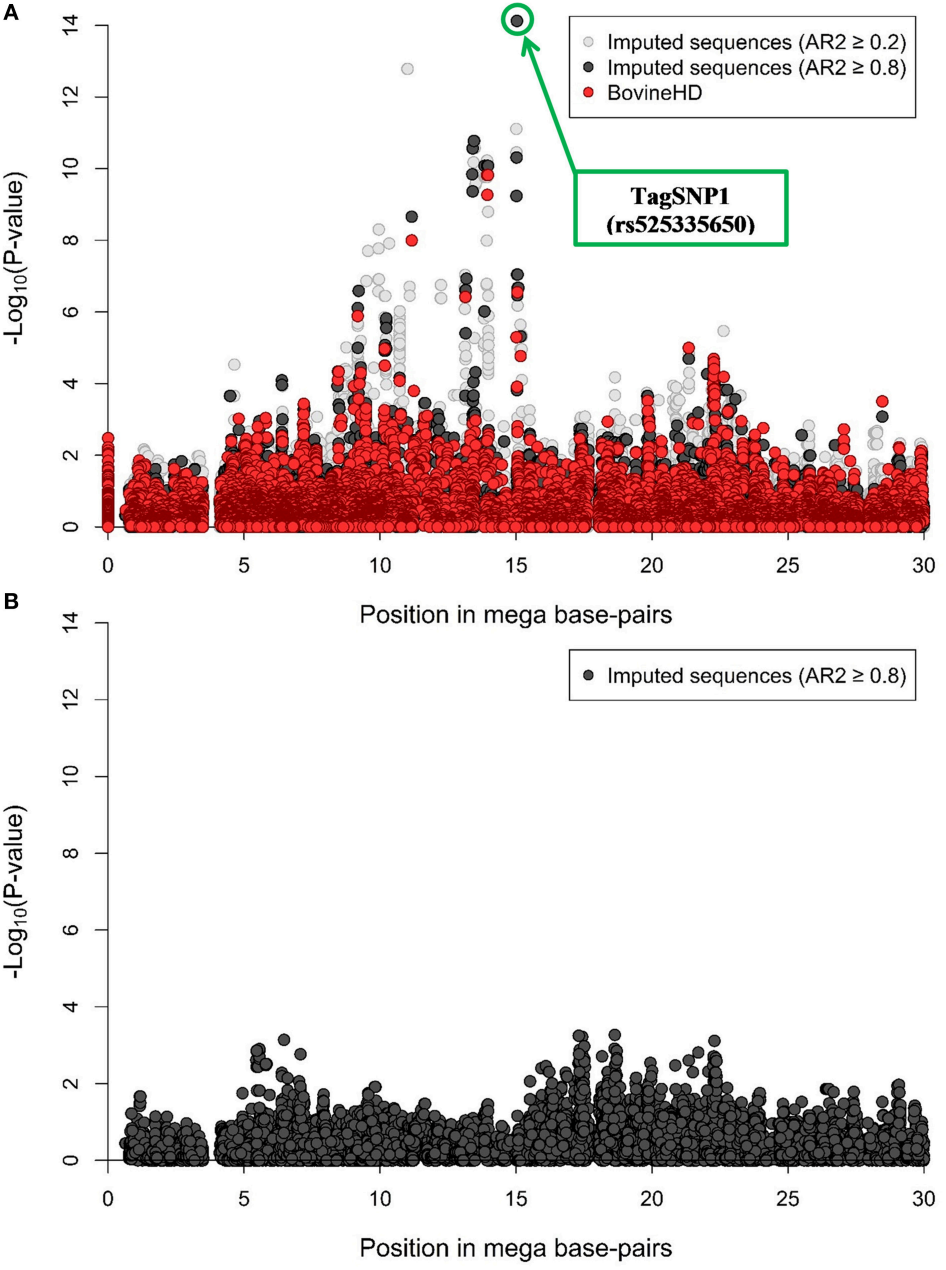
**Region-wide association study (RWAS) with non-coagulating (NC) milk in 382 Swedish Red cows**. **(A)** RWAS based on 137,949 polymorphic imputed variants overlaid with the BovineHD genotypes for half of BTA18. In light gray, imputed variants with accuracy of imputation (AR2) ≥ 0.2. In black, imputed variants with AR2 ≥ 0.8. “TagSNP1” as most significant association. **(B)** RWAS after correcting for TagSNP1. In black, imputed variants with AR2 ≥ 0.8 (N = 80,206 variants).

A RWAS based on imputed sequences was run for half of BTA18, which corresponds to a genomic region of 30 MBP running from position 0 on bovine genome built UMD 3.1. Throughout this region, a total of 205 variants were significantly associated with NC milk at −Log_10_ (*P*-value) > 6 and imputed with AR2 ≥ 0.8 (Supplementary Table 1). The most significant variants were one indel and two SNP. The indel was rs385975260 occurring at 15.03 MBP, and was imputed with AR2 = 0.87. The first SNP was rs525335650 located at 15.03 MBP, and was imputed with AR2 = 0.87. The second SNP was rs379827811 located at 15.04 MBP, and was imputed with AR2 = 0.42. These two SNP and one indel are in perfect LD with each other. We chose rs525335650 among these three imputed variants, since it was the best imputed variant, and tagged it as TagSNP1 (Figure 1A).

After including TagSNP1 as a fixed effect in model 1, a total of 80,206 variants with an AR2 ≥ 0.8 were re-analyzed. We re-analyzed these 80,206 imputed variants instead of the 137,949 imputed variants to reduce potential false-positive associations with NC milk caused by imputation errors. After accounting for TagSNP1 in model 1 as fixed effect, no remaining associations were found (Figure 1B).

### Haplotype analyses

**Figure 2:**
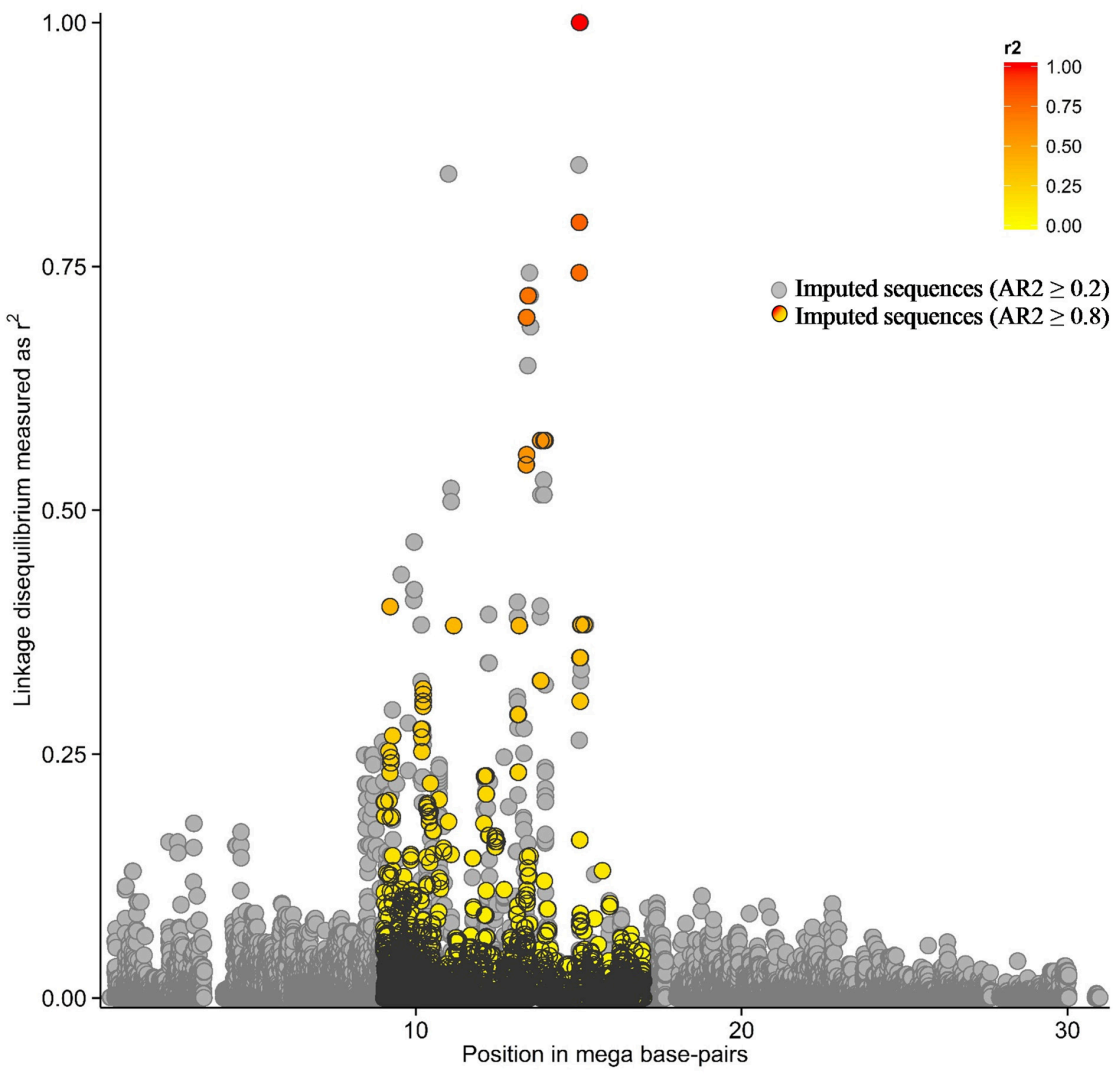
**Linkage disequilibrium in the QTL region**. In the colored region, pairwise linkage disequilibrium as the squared correlation between the most significant association, “TagSNP1,” and all other markers. In light gray, imputed variants with accuracy of imputation (AR2) ≥ 0.2. In black, imputed variants with AR2 ≥ 0.8.

**Figure 3:**
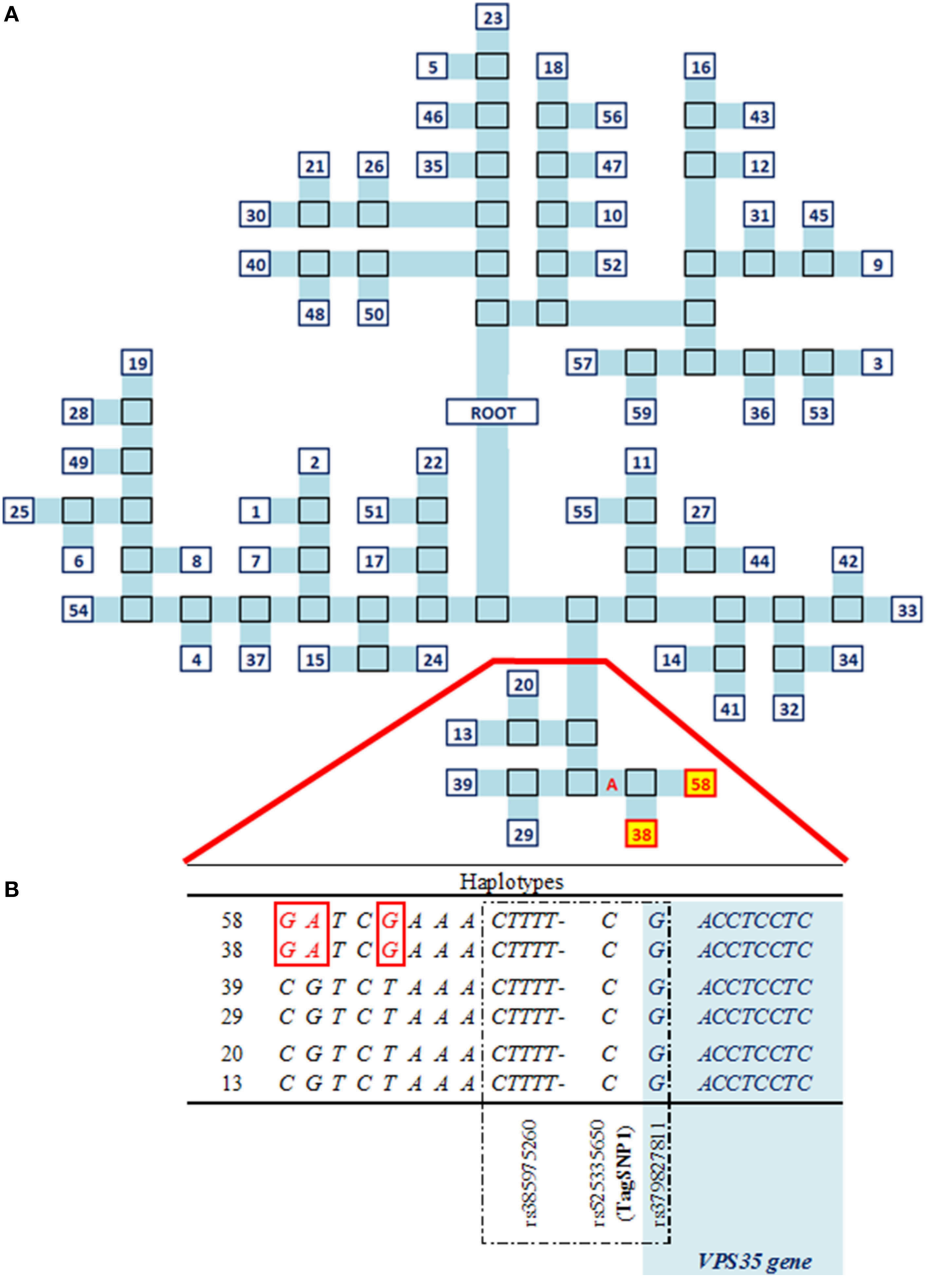
**Haplotypes analyses characterizing the QTL region in SR cows**. **(A)** Phylogenetic tree of the 59 unique haplotypes, numbered in blue. In light blue, a branch of the tree. In black borders, bipartitions. In red and yellow, significant haplotypes at *P* < 0.05. **(B)** Relevant part of the sequences of significant vs. other haplotypes. In red, differences between haplotypes. Dashed in black, strongest associations including TagSNP1. In light blue, the *VPS35* gene.

A total of 129 SNP plus 17 indels in LD with TagSNP1 (Figure 2) were combined into 59 haplotypes. These 59 haplotypes were the basis to build a phylogenetic tree, for which each branch represented one unique haplotype segregating in the SR cow population (Figure 3A). The iterative inference of haplotypes using TreeScan occurred by, for example, cutting the phylogenetic tree in two parts at branch “A,” where haplotypes 38 and 58 were grouped in one part, while all other haplotypes were grouped in the other part. The parts were then tested against each other. After all branches of the tree were tested, associations with NC milk were: branch “A” at *P* = 0.002; haplotype 38 at *P* = 0.03; and, haplotype 58 at *P* = 0.03 (Figure 3A). Next, we scrutinized in depth the sequences of haplotypes 38 and 58, and we found they have three SNP in common. When comparing haplotypes 38 and 58 with haplotypes 13, 20, 29, and 39 (Figure 3B), haplotypes 38 and 58 differed from the other haplotypes at these exact same three SNP. Interestingly, these three SNP shared by haplotypes 38 and 58 are quite close to our TagSNP1 (Figure 3B).

### Bioinformatics and candidate genes

According to Ve!P, the 129 SNP plus the 17 indels, which included our TagSNP1, were distributed as follows: 32% of intron variants; 26% of downstream gene variants, 25% of upstream gene variants; 12% of intergenic variants; 4% of 3′ UTR variants; 1% of synonymous variants, and 1% of missense variants. In summary, 67% of these 129 SNP plus 17 indels were synonymous variants without changes to the encoded amino acids. The remaining 33% were missense variants with changes in one or more bases to the encoded amino acid.

In addition, Ve!P showed that our QTL region on BTA18 contains seven genes (Table 3), of which one is a validated gene and six are provisional genes. These seven genes are: validated carbonic anhydrase VA, mitochondrial (***CA5A***) gene; BTG3 associated nuclear protein (***BANP***) gene; cytochrome b-245, alpha polypeptide (***CYBA***) gene; mevalonate (diphospho) decarboxylase, mRNA (***MVD***) gene; snail family zinc finger 3 (***SNAI3***) gene; ring finger protein 166 (***RNF166***) gene; and, vacuolar protein sorting 35 homolog, mRNA (***VPS35***) gene. In addition, the *CA5A* gene is located within a copy number variation.

**Table 3:** **Details about candidate genes identified in the QTL region**.

**Genes**	**Identifier**	**Location**	**Numbers of variants**
*CA5A*	ENSBTAG00000010151	chr18: 13,356,215–13,445,854	8
*BANP*	ENSBTAG00000023745	chr18: 13,425,303–13,493,366	3
*CYBA*	ENSBTAG00000003895	chr18: 13,931,107–13,938,075	40
*MVD*	ENSBTAG00000012059	chr18: 13,938,827–13,945,489	72^*^
*SNAI3*	ENSBTAG00000017528	chr18: 13,958,995–13,964,622	36^**^
*RNF166*	ENSBTAG00000020942	chr18: 13,969,303–13,977,633	3
*VPS35*	ENSBTAG00000002493	chr18: 15,038,821–15,066,463	2

* 40 of these 72 variants in the MVD gene overlap with variants in the CYBA gene. These are: 26 downstream variants in the MVD gene corresponding to 16 introns, 1 synonymous, and 9 upstream variants in the CYBA gene; and seven 3′ UTR, 1 synonymous, 5 intron, and 1 missense variants in the MVD gene corresponding to upstream variants in the CYBA gene

** *5 of these 36 hits are downstream gene variants in the SNAI3 gene that correspond to upstream gene variants in the RNF166 gene*.

The genomic position of the three strongest associations with NC milk on BTA18 are shown in Supplementary Figure 3A. Of these associations, rs379827811 is an intron variant in the *VPS35* gene. According to Ve!P, rs379827811 is upstream to 14 missense variants, 1 synonymous variant, 1 stop gained variant and 1 splice region variant (Supplementary Figure 3B).

The three SNP shared by haplotypes 38 and 58 identified in the haplotype analyses are intergenic variants located between 20.5 and 31.2 kilo base-pairs (**kbp**) downstream to the *VPS35* gene.

## Discussion

In the present study, we used the same phenotypes and BovineHD genotypes as in Gustavsson et al. (2014a) to perform a GWAS with NC milk, and we further fine-mapped a genomic region on half of BTA18 using imputed sequences. This genomic region is distributed over seven MBP on BTA18, and is strongly associated with NC milk. At least one QTL could be fine-mapped using imputed sequences. In addition, we conducted haplotype analyses to disentangle the occurrence of multiple QTL in strong LD within this region. At last, we identified potential candidate genes within this QTL region.

### GWAS on BovineHD genotypes

The GWAS on BovineHD genotypes showed significant associations with NC milk distributed over seven mega base-pairs (**MBP**) on BTA18 (Table 1). These seven MBP explain large fractions of the phenotypic variation in NC milk, ranging from 7 to 11%. Tyrisevä et al. (2008) performed a genome scan to map non-coagulation of milk in 477 genotyped FAY cows. Their study used microsatellite markers and identified a QTL located around 17 MBP on BTA18. Their QTL is very close to the seven MBP region identified in our study. The methodology used by Tyrisevä et al. (2008) is different from the present study. It is important to note that the study by Tyrisevä et al. (2008) is based on a linkage study within sire families with pooled DNA of cows with extreme phenotypes, and our study is based on an association analysis of genotyped cows with scored phenotypes. Both methodologies have the common goal of pointing out the potential candidate genes associated with a trait of interest, and, despite the differences between both studies, a similar genomic region was associated with NC milk.

Eleven significant associations found by our GWAS were in agreement with associations found by the GWAS of Gregersen et al. (2015), who studied MCP properties but not NC milk. This agreement occurred with the following two traits: rennet gel strength measured 30 min after chymosin addition (**G'30**), and rennet coagulation time (**CTrennet**). For G'30, associations agreed on BTA1, BTA13, BTA18, and BTA22. More specifically, these associations were: four SNP located between 70.75 and 70.90 MBP on BTA1; five SNP located between 58.10 and 58.14 MBP on BTA13; one SNP at 13.13 MBP on BTA18; and one SNP located at 19.35 MBP on BTA22. The strong negative genetic correlation between NC milk and G'30 (-0.82; Gustavsson et al., 2014a) is likely to explain the agreement of results between both studies regarding G'30. For CTrennet, associations agreed on BTA18, and these were: one SNP located at 11.16 MBP, and one SNP located at 11.65 MBP. Gregersen et al. (2015) used (log-transformed) CTrennet in their GWAS, whereas we analyzed NC milk, a trait derived from CTrennet. Despite the use of different but CTrennet-related traits, it was unexpected to find only two associations in agreement between both studies. A reason for this little agreement might be caused by our approach to analyze NC milk, which dealt with the right-censored nature of coagulation time in a more suitable way (Cecchinato and Carnier, 2011).

An important aspect of our GWAS on BovineHD genotypes was the analyses of NC milk as a normally distributed trait despite its binary nature. Cecchinato and Carnier (2011) were the first authors to suggest this approach because NC milk samples have been consistently excluded from most analyses when observed (e.g., Ikonen et al., 2004; Gregersen et al., 2015). Cecchinato and Carnier (2011) showed that statistical models have difficulties to correctly account for NC milk, and suggested to score NC milk as a binary trait and include it as a normally distributed trait in linear mixed models. This option allows for analyses of NC milk without the exclusion of information. Following this approach, Gustavsson et al. (2014a) included NC milk as a binary trait in their analyses, and estimated genetic parameters for rennet-induced coagulation properties in SR cows. In addition, the inclusion of NC milk as a binary trait in our study could be one of the reasons why little overlap was found with the study by Gregersen et al. (2015) regarding CTrennet.

Besides their GWAS, Gregersen et al. (2015) found a suggestive QTL for the log-transformed G'30 trait by haplotypes analyses. This suggestive QTL was found in the interval located between 11.65 and 22.34 MBP on BTA18. Although, not significant in their study, this suggestive QTL interval is in agreement with 9 out of 10 of our most significant SNP associated with NC milk on BTA18 (Table 1). In addition, the top SNP indicated by Gregersen et al. (2015) at 11.16 MBP is among our most significant SNP associated with NC milk.

Breeding for higher protein content in SR cows might lead to problems in the foreseeable future, suggested by the moderate, yet unfavorable genetic correlation between NC milk and protein content (Gustavsson et al., 2014a). Our main goal was to disentangle the effects of genetic variants of milk protein fractions from other genetic variants associated with NC milk. For this reason, we included a multi-locus genotype that combined the genetic variants of the main milk protein fractions (i.e., αs1-β-κ-CN; “CNcluster”) in our model. Bittante et al. (2012) reviewed the most important genetic factors that affect MCP, indicating that MCP, including NC milk, are strongly influenced by variable proportions, and genetic variants of milk protein fractions (especially of κ-CN). These milk protein fractions, mainly representing caseins, are encoded on BTA6 and thus, the recombination among alleles is small (Bittante et al., 2012). In contrast, Tyrisevä et al. (2008) did not find significant associations between NC milk and the casein loci. In the present study, the casein loci were included as part of the design of our GWAS with NC milk, resulting in significant associations that are independent from the casein loci. This means that genes found by our study represent a new set of genes compared with the genes of the casein loci known to influence the prevalence of NC milk (e.g., Jensen et al., 2012; Gustavsson et al., 2014b).

### Imputation

Imputation of SR cows was quite challenging because most of the variants were poorly imputed at sequence level when directly using the 429 WGS as reference population. As pointed out by Bouwman and Veerkamp (2014), breed-specific variants are best imputed by using a large single-breed reference population. This suggestion would mean that only 33 out of the 429 WGS would be of interest to impute our 382 SR cows to sequence level. The challenge of imputing a small breed like SR was overcome by running three different scenarios of imputation, and each time with a different reference population. The genotype that had the best imputation accuracy across the three scenarios was selected as the best-imputed genotype. The average accuracies of imputation using our approach were 0.79 for variants imputed with AR2 ≥ 0.2, and 0.93 for variants imputed with AR2 ≥ 0.8. While this is a slightly *ad-hoc* approach, there was good concordance between the three imputation scenarios and our subsequent focus on variants with AR2 ≥ 0.8 adds further rigor to our analyses.

### RWAS on imputed sequences for half of BTA18

The RWAS on imputed sequences for half of BTA18 revealed many significant associations with NC milk (Supplementary Table 1). One of our three strongest associations, TagSNP1, explained almost 34% of the genetic variation and 14% of the phenotypic variance in NC milk (Figures 1A,B). This large fraction of genetic variance explained by TagSNP1 is independent of the casein loci located on BTA6. Altogether, these findings strongly suggest the existence of at least one causal variant in our focus region distributed over seven MBP associated with NC milk. It might be plausible that one causal variant, i.e., 1 QTL is associated with NC milk in our focus region, although we cannot exclude the presence of multiple QTL in strong LD associated with NC milk in our focus region. Similar findings were found by Daetwyler et al. (2014) and Sahana et al. (2014). In their GWAS with imputed sequences, the considerable number of significant variants closely linked to each other increased the complexity of identifying a causal variant. In our study, we performed haplotype analyses to answer whether one or multiple QTL were present in the seven MBP.

### Haplotype analyses

Among the many advantages of haplotype over single-variant analyses (Balding, 2006), two of them are: (a) haplotype analyses naturally account for the correlated structure between variants because all the genetic variation in a population is transmitted from parent to offspring through haplotypes (Clark, 2004); and (b) haplotype analyses reduce the number of parameters tested in association studies as compared with single-variant analyses (e.g., Clark, 2004; Balding, 2006). In contrast, a “tagging” strategy would reduce the power gained from using haplotypes *per se* (Balding, 2006). In our study, this limitation was dealt with by using the TreeScan approach (Templeton et al., 2005). TreeScan considered two aspects simultaneously: the correlated structure of variants closely linked to each other, and the origin of this haplotype in the population through a phylogenetic tree. Using the TreeScan approach, 2 out of the 59 haplotypes were found to be associated with NC milk in our QTL region (Figure 3A). The two significant haplotypes had three SNP in common, and these SNP are located from 13.7 to 24.4 kbp apart from TagSNP1 (Figure 3B). These findings support the presence of one QTL influencing NC milk in our focus region. Nonetheless, the task of identifying the causal variant remains challenging. According to Vasemägi and Primmer (2005), when an association between TagSNP1 and the causal variant is found, other linked associations can be responsible for the variation in the trait of interest. This might be our case since TagSNP1 was one out of three closely linked variants strongly associated with NC milk.

### Bioinformatics and candidate genes

Our three strongest association with NC milk are composed of one indel and two SNP. One of these two SNP (rs379827811) is an intron variant located between 15.04 MBP within the *VPS35* gene (Figure 3B, Supplementary Figures 3A,B). In humans, the *VPS35* gene is a component of the retromer complex that mediates endosome-to-Golgi retrieval of membrane proteins such as the cation-independent mannose 6-phosphate receptor. According to Malik et al. (2015), cargo-selective sorting is important for the correct sub-cellular destination of membrane proteins. The retromer complex mediated by VPS35 gene seems to promote the recycling of specific membrane proteins, such as β2- adrenergic receptor and the glucose transporter GLUT1, directly back to plasma membrane (Seaman et al., 2013). It is important to mention that GLUT1 is the major glucose transporter in the basal membrane of epithelial cells and, in the mice mammary gland, its expression was increased when greater demand for glucose for the synthesis of lactose was needed (Anderson et al., 2007). If the recycling mechanism of the retromer complex is defective, it is possible that not enough membrane proteins are recycled, and in turn, are not available for milk synthesis.

A mutation in the *VPS35* gene has been associated with Parkinson's disease (Zavodszky et al., 2014). In mice-models for Parkinson's disease, a *VPS35* deficiency could contribute to retinal ganglion neuro-degeneration, leading to the blindness of many retinal degenerative disorders (Liu et al., 2014). In addition, Lemay et al. (2013) shows that the *VPS35* gene is expressed throughout lactation in humans, which include colostrum, transitional, and mature milk, after they sequenced the mRNA found in milk fat layer. In Arabidopsis, the *VPS35* gene has been associated with protein sorting and is involved in the plant growth and leaf senescence (Yamazaki et al., 2008). In addition, Munch et al. (2015) shows that a dysfunction in the *VPS35* gene can contribute to immune-associated cell death in Arabidopsis. In cattle, Lemay et al. (2009) classified the mammary gene sets according to their condition and their developmental specific-stage, and showed that the *VPS35* gene belonged to the mammary gene sets of pre-parturient and of lactating cows. The VPS35 gene has not been associated to NC milk yet.

## Conclusions

The GWAS on BovineHD genotypes found significant associations with NC milk distributed over seven MBP on BTA18 for SR cows. These seven MBP contained 14 SNP that explained from 7 to 11% of phenotypic variation in NC milk. This large proportion of explained phenotypic variance is independent of the casein loci. To further characterize these seven MBP, we ran a region-wide association study with imputed sequences. The significance of the associations increased from −Log_10_(*P*-value) = 10.18 on BovineHD genotypes to −Log_10_(*P*-value) = 14.12 on imputed sequences. NC milk in SR cows was influenced by at least one QTL within these seven MBP. A haplotype analyses identified 2 haplotypes that differed from the other 57 haplotypes at three SNP. These three SNP were located near to the strongest association identified by the region-wide association study with imputed sequences. For BTA18, haplotype analyses support the existence of one QTL underlying NC milk in SR cows. A candidate gene of interest is the *VPS35* gene, for which one of our strongest association is an intronic SNP in this gene. It has been suggested that the VPS35 gene is involved in the recycling of specific membrane proteins, such as β2- adrenergic receptor and the glucose transporter GLUT1. The *VPS35* gene belongs to the mammary gene sets of pre-parturient and of lactating cows, and has not been associated to NC milk yet.

## Ethics statement

The study was approved by the Swedish Board of Agriculture, Gothenburg ethical committee, on 22 March 2010 (Registration number 51-2010).

## Author contributions

MG and MP coordinated the data collection and analysis of milk samples. MG, MP, WF, and DJ designed and supervised the study. SD, DJ, and WF analyzed the data and interpreted the results. SD, MG, DJ, MP, WF wrote the manuscript. All authors revised and accepted the final version of the manuscript.

### Conflict of interest statement

The authors declare that the research was conducted in the absence of any commercial or financial relationships that could be construed as a potential conflict of interest.

## Abbreviations

AR2: beagle's accuracy of imputation
BovineHD: 777,963 SNP genotypes
BTA: *Bos taurus* autosome
CN: caseins
CTrennet: rennet coagulation time
FAY: Finnish Ayrshire
G'30: rennet gel strength measured 30 min after chymosin addition
GWAS: genome-wide association study
LD: linkage disequilibrium
MAF: minor allele frequency
MBP: mega base-pair
MCP: milk coagulation properties
NC: non-coagulating
QTL: quantitative trait loci
RWAS: region-wide association study
SR: Swedish Red
TagSNP1: most significant association retrieved from RWAS
Ve!P: variant effect predictor
WGS: whole-genome sequences.
