# Supplementary material for: Identification of QTL on Chromosome 18 Associated with Non-Coagulating Milk in Swedish Red Cows
Source: Front Genet. 2016 Apr 15;7:57. doi: 10.3389/fgene.2016.00057 (PMC4832587; doi:10.3389/fgene.2016.00057)
Supplement: Supplementary file 1 [file Image1.PDF]

Supplementary Material

Identification of QTL on chromosome 18 associated with non-coagulating milk in Swedish Red cows

Sandrine I. Duchemin*, Maria Glantz, Dirk-Jan de Koning1, Marie Paulsson, and Willem F. Fikse

*** Correspondence:** Corresponding Author: sandrine.duchemin@wur.nl

1 Supplementary Figures

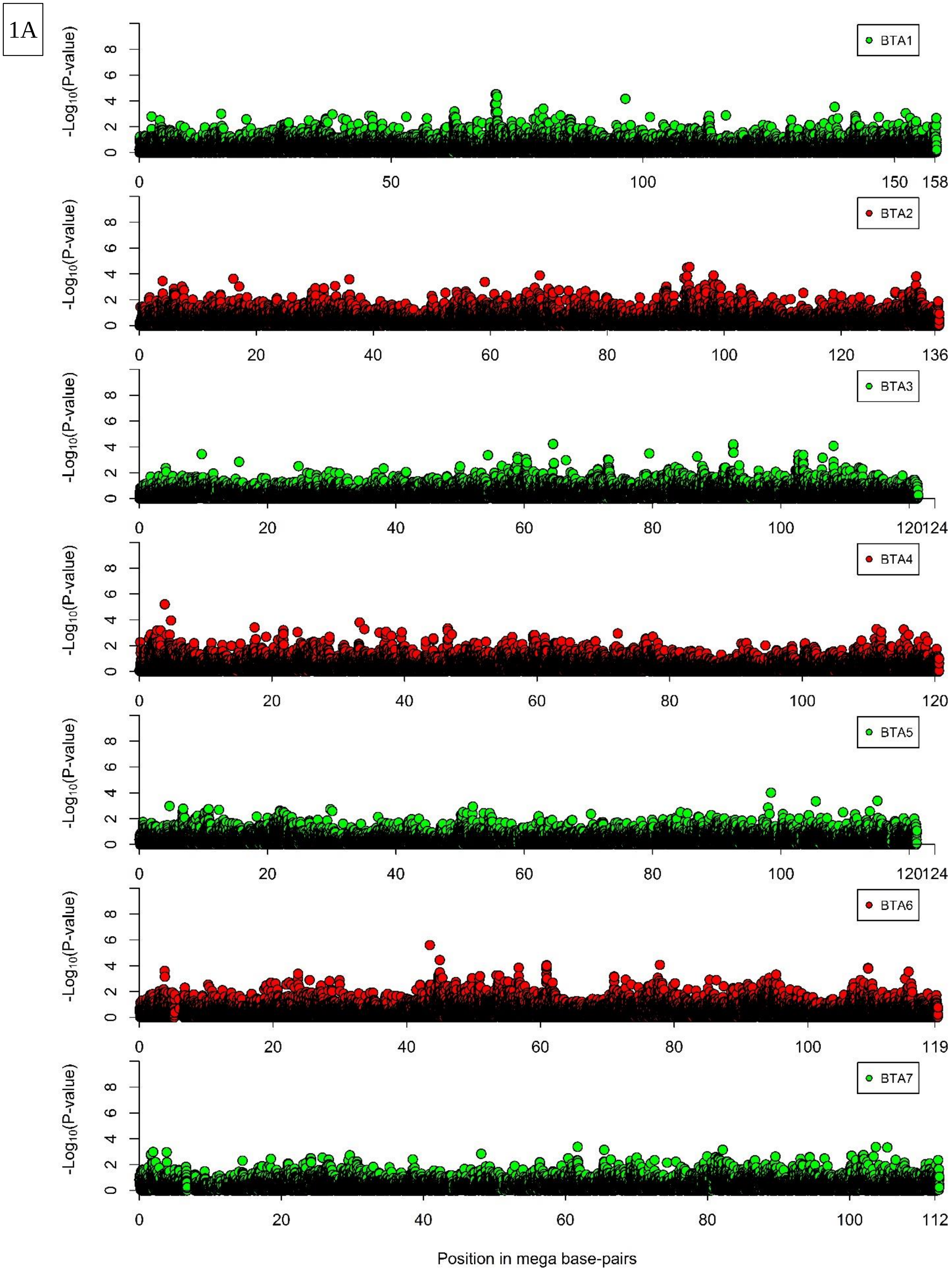

1A
BTA1
BTA2
BTA3
BTA4
BTA5
BTA6
BTA7
-Log10(P-value)
0
2
4
6
8
50
100
150
158
20
40
60
80
120
136
120124
119
112
Position in mega base-pairs

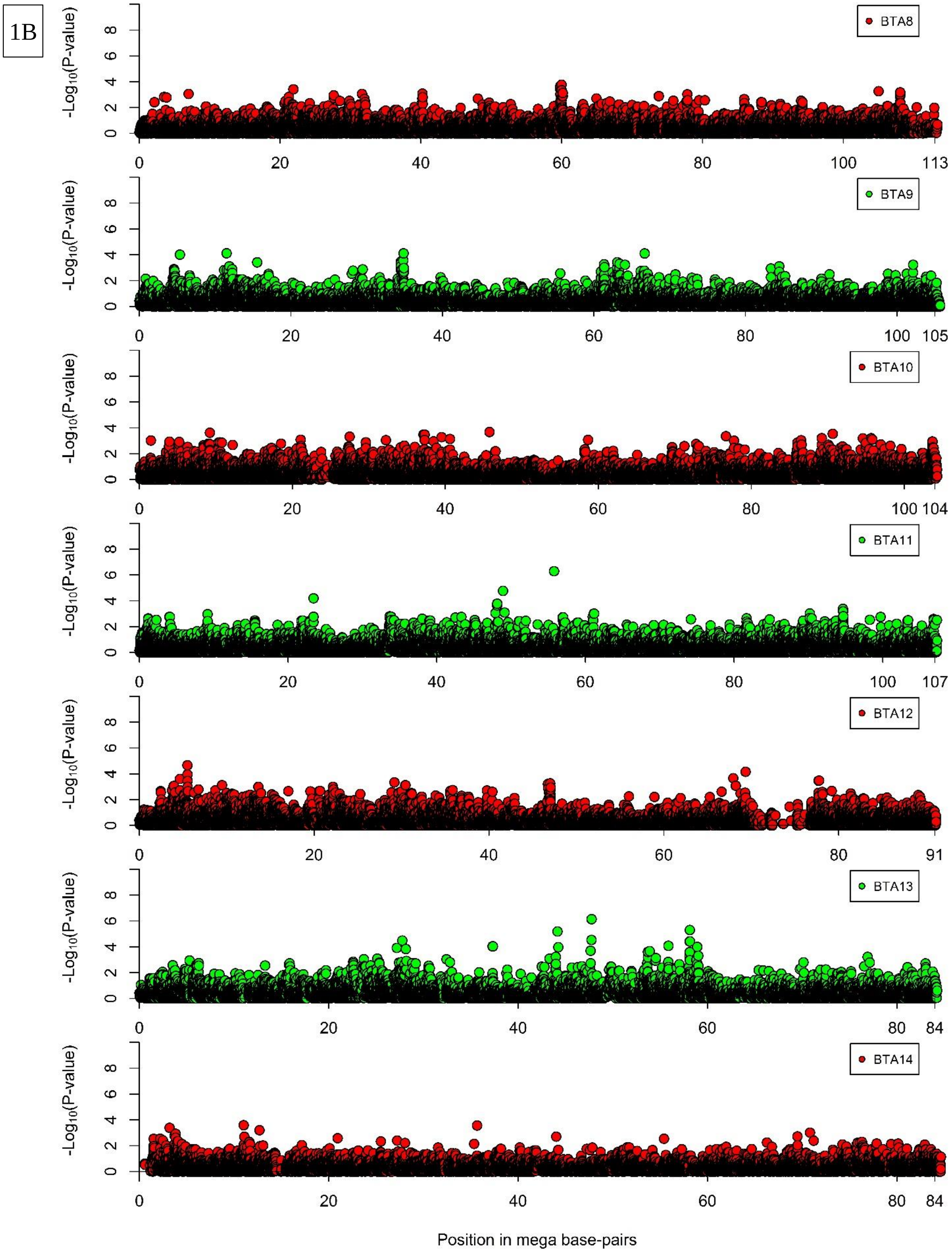

1B
BTA8
BTA9
BTA10
BTA11
BTA12
BTA13
BTA14
-Log10(P-value)
Position in mega base-pairs

1C

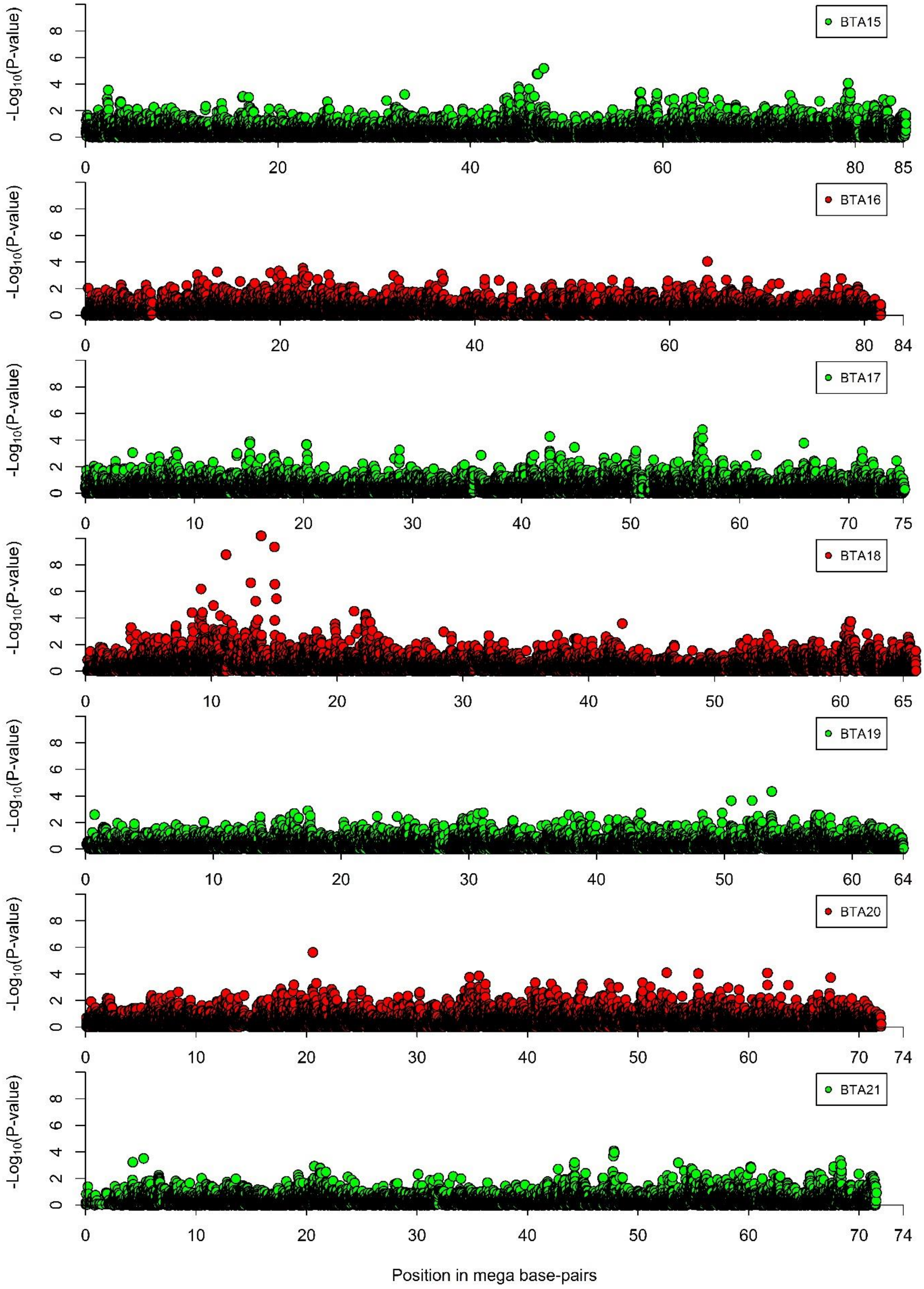

BTA15
BTA16
BTA17
BTA18
BTA19
BTA20
BTA21
-Log10(P-value)
Position in mega base-pairs

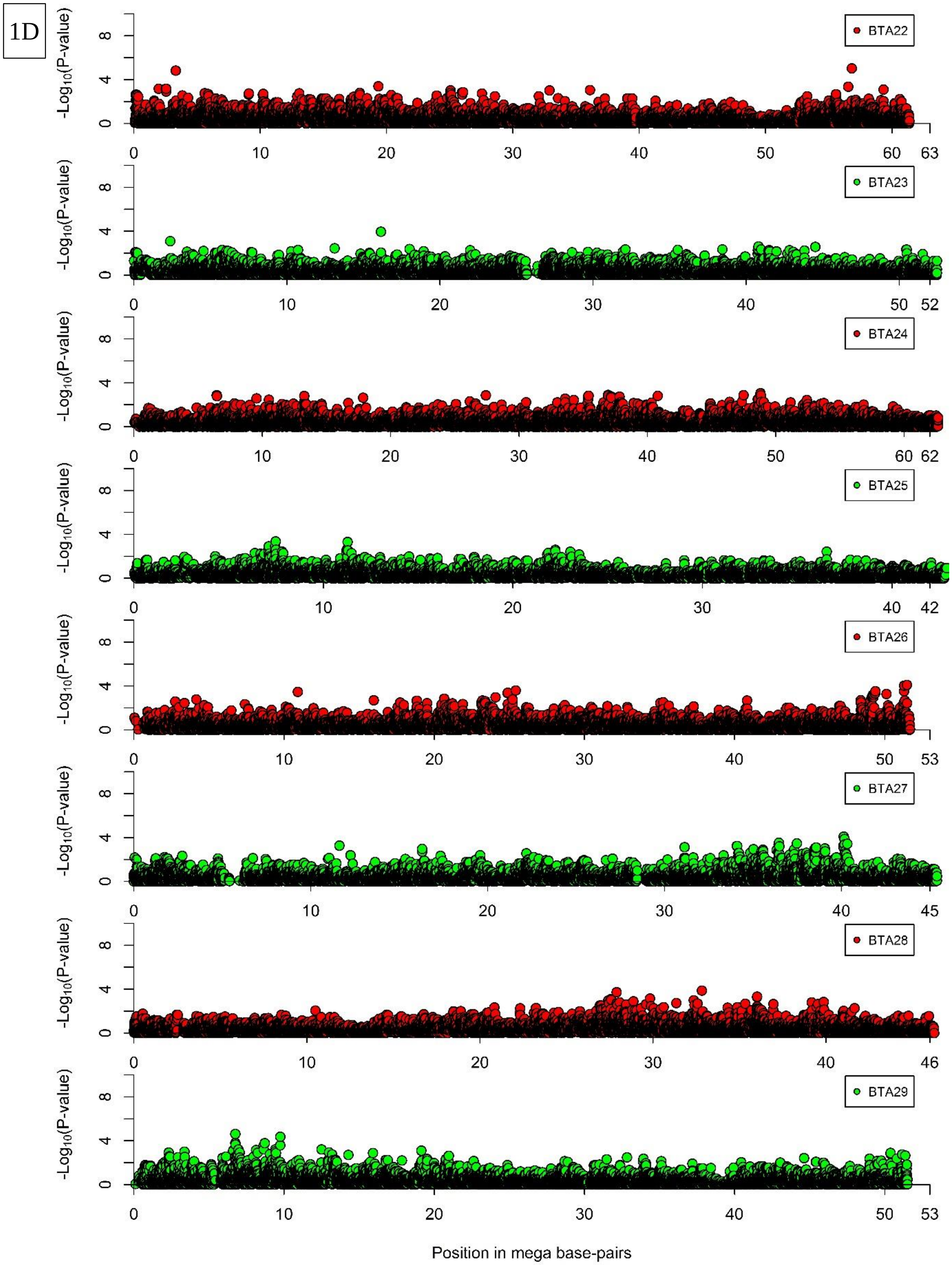

Supplementary Figure 1. Genome-wide association study using 777,963 SNP genotypes affecting non-coagulating milk in Swedish Red cows. **(A)** Results for BTA1 through BTA7. **(B)** Results for BTA8 through BTA14. **(C)** Results for BTA15 through BTA21. **(D)** Results for BTA22 through BTA29.
