# Supplementary material for: Identification of QTL on Chromosome 18 Associated with Non-Coagulating Milk in Swedish Red Cows
Source: Front Genet. 2016 Apr 15;7:57. doi: 10.3389/fgene.2016.00057 (PMC4832587; doi:10.3389/fgene.2016.00057)
Supplement: Supplementary file 2 [file Image2.PDF]

Supplementary Material

Identification of QTL on chromosome 18 associated with non-coagulating milk in Swedish Red cows

Sandrine I. Duchemin*, Maria Glantz, Dirk-Jan de Koning1, Marie Paulsson, and Willem F. Fikse

*** Correspondence:** Corresponding Author: sandrine.duchemin@wur.nl

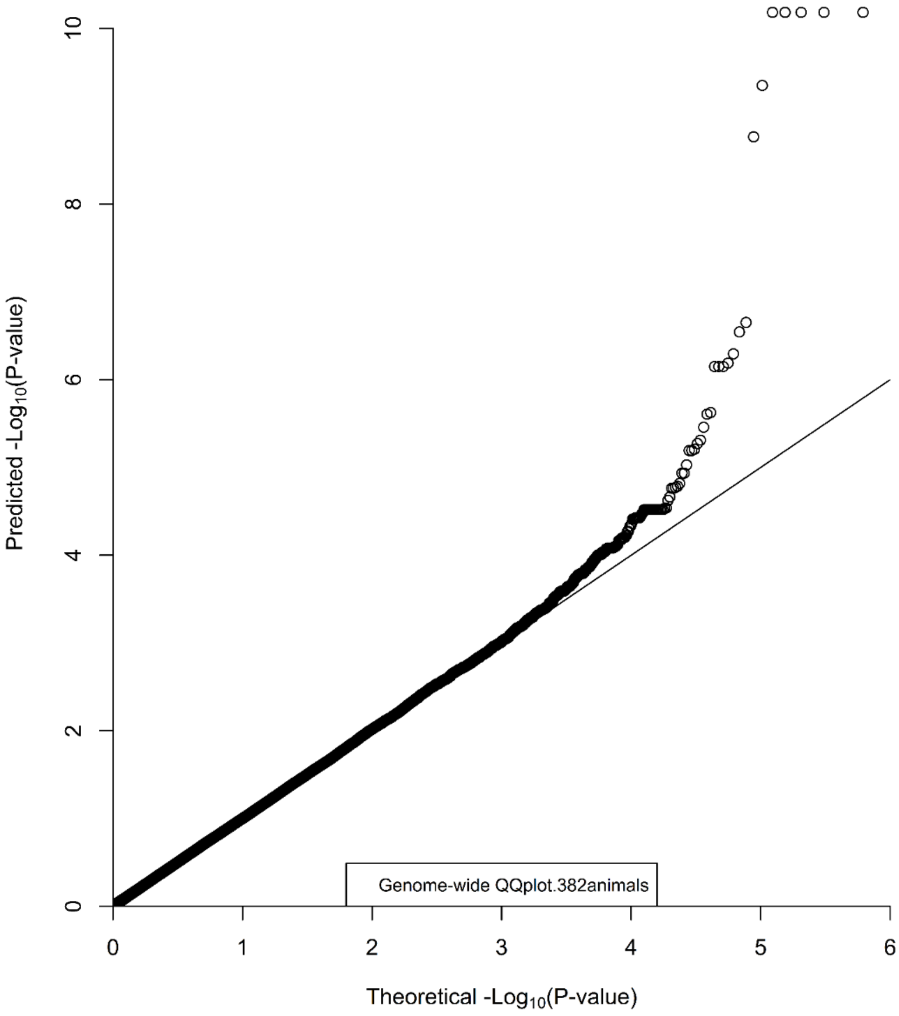

Supplementary Figure 2. Genome-wide QQ-Plot for the GWAS with NC milk based on 777,963 SNP genotypes and 382 Swedish Red Cows
