# Supplementary material for: Identification of QTL on Chromosome 18 Associated with Non-Coagulating Milk in Swedish Red Cows
Source: Front Genet. 2016 Apr 15;7:57. doi: 10.3389/fgene.2016.00057 (PMC4832587; doi:10.3389/fgene.2016.00057)
Supplement: Supplementary file 3 [file Image3.PDF]

Supplementary Material

Identification of QTL on chromosome 18 associated with non-coagulating milk in Swedish Red cows

Sandrine I. Duchemin*, Maria Glantz, Dirk-Jan de Koning1, Marie Paulsson, and Willem F. Fikse

*** Correspondence:** Corresponding Author: sandrine.duchemin@wur.nl

3A

3B

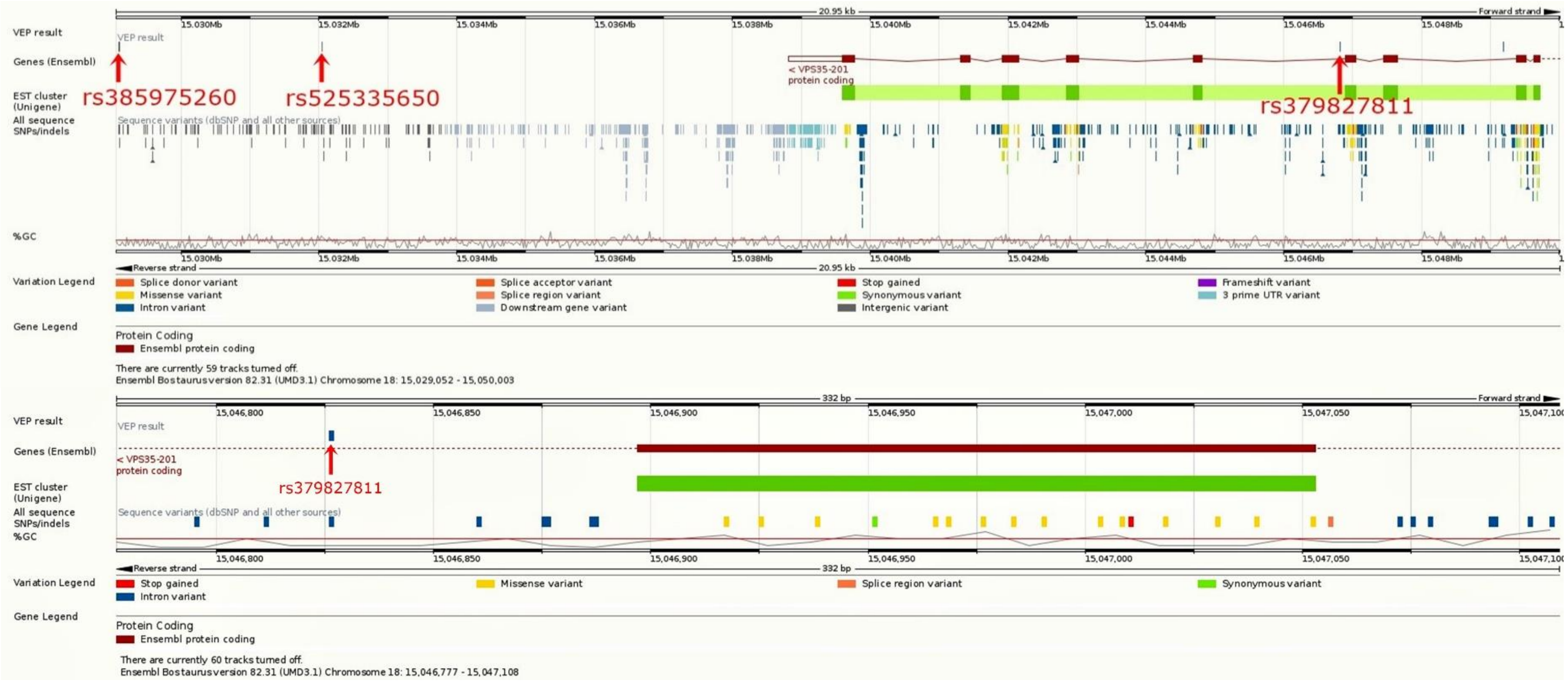

Supplementary Figure 3. Views from Ensembl (http://www.ensembl.org) of strongest associations. **(A)** Genomic location of rs385975260, rs525335650 (TagSNP1), and rs379827811. **(B)** rs379827811 as intron variant to the *VPS35* gene.
