# Supplementary material for: Identification of QTL on Chromosome 18 Associated with Non-Coagulating Milk in Swedish Red Cows
Source: Front Genet. 2016 Apr 15;7:57. doi: 10.3389/fgene.2016.00057 (PMC4832587; doi:10.3389/fgene.2016.00057)
Supplement: Supplementary file 4 [file Table1.docx]

Supplementary Material

**Identification of QTL on chromosome 18 associated with non-coagulating milk in Swedish Red cows**

Sandrine I. Duchemin*, Maria Glantz, Dirk-Jan de Koning1, Marie Paulsson, and Willem F. Fikse

*** Correspondence:** Corresponding Author: [sandrine.duchemin@wur.nl](mailto:sandrine.duchemin@wur.nl)

**Supplementary Table 1. Region-wide association study** - list of most significant variants associated with non-coagulating (**NC**)^†^ milk in Swedish Red cows

| Chromosome | Name of variant | -Log_10_  (Pvalue) | AR2^§^ | $\sigma_{marker}^{2}$^¢^ | $\frac{\sigma_{marker}^{2}}{\sigma_{p}^{2}}$^*^ |
| --- | --- | --- | --- | --- | --- |
| 18 | 18:9179338 | 6.11 | 0.80 | 0.01 | 0.07 |
| 18 | 18:9179437 | 6.11 | 0.82 | 0.01 | 0.07 |
| 18 | 18:9179455 | 6.11 | 0.83 | 0.01 | 0.07 |
| 18 | 18:9179462 | 6.11 | 0.83 | 0.01 | 0.07 |
| 18 | 18:9179471 | 6.11 | 0.81 | 0.01 | 0.07 |
| 18 | 18:9179491 | 6.11 | 0.82 | 0.01 | 0.06 |
| 18 | 18:9179500 | 6.11 | 0.82 | 0.01 | 0.06 |
| 18 | 18:9179561 | 6.11 | 0.91 | 0.01 | 0.06 |
| 18 | 18:9179563 | 6.11 | 0.91 | 0.01 | 0.06 |
| 18 | 18:9179722 | 6.11 | 1.00 | 0.01 | 0.07 |
| 18 | 18:9179819 | 6.11 | 0.99 | 0.01 | 0.07 |
| 18 | 18:9179826 | 6.11 | 1.00 | 0.01 | 0.07 |
| 18 | 18:9179834 | 6.11 | 1.00 | 0.01 | 0.07 |
| 18 | 18:9180145 | 6.11 | 0.99 | 0.01 | 0.07 |
| 18 | 18:9180426 | 6.11 | 0.99 | 0.01 | 0.07 |
| 18 | 18:9180513 | 6.11 | 0.99 | 0.01 | 0.07 |
| 18 | 18:9180543 | 6.11 | 0.99 | 0.01 | 0.07 |
| 18 | 18:9180617 | 6.11 | 0.99 | 0.01 | 0.07 |
| 18 | 18:9180637 | 6.11 | 0.99 | 0.01 | 0.07 |
| 18 | 18:9181238 | 6.11 | 0.99 | 0.01 | 0.07 |
| 18 | 18:9181315 | 6.11 | 0.99 | 0.01 | 0.07 |
| 18 | 18:9181629 | 6.11 | 0.99 | 0.01 | 0.07 |
| 18 | 18:9181646 | 6.11 | 0.99 | 0.01 | 0.07 |
| 18 | 18:9182405 | 6.11 | 0.81 | 0.01 | 0.08 |
|  |  |  |  |  |  |
| Chromosome | Name of variant | -Log_10_  (Pvalue) | AR2^§^ | $\sigma_{marker}^{2}$^¢^ | $\frac{\sigma_{marker}^{2}}{\sigma_{p}^{2}}$^*^ |
| 18 | 18:9214353 | 6.59 | 0.85 | 0.01 | 0.08 |
| 18 | 18:9215169 | 6.59 | 0.96 | 0.01 | 0.07 |
| 18 | 18:9215335 | 6.59 | 0.86 | 0.01 | 0.08 |
| 18 | 18:9215376 | 6.59 | 0.96 | 0.01 | 0.07 |
| 18 | 18:9215787 | 6.59 | 0.96 | 0.01 | 0.07 |
| 18 | 18:9215948 | 6.59 | 0.96 | 0.01 | 0.07 |
| 18 | 18:9216194 | 6.59 | 0.96 | 0.01 | 0.07 |
| 18 | 18:11166809 | 8.66 | 1.00 | 0.01 | 0.09 |
| 18 | 18:13136070 | 6.61 | 0.83 | 0.01 | 0.07 |
| 18 | 18:13136171 | 6.61 | 1.00 | 0.01 | 0.07 |
| 18 | 18:13137293 | 6.61 | 0.95 | 0.01 | 0.07 |
| 18 | 18:13138676 | 6.61 | 0.95 | 0.01 | 0.07 |
| 18 | 18:13142955 | 6.61 | 0.90 | 0.01 | 0.07 |
| 18 | 18:13145923 | 6.61 | 0.90 | 0.01 | 0.07 |
| 18 | 18:13146013 | 6.61 | 0.90 | 0.01 | 0.07 |
| 18 | 18:13146020 | 6.61 | 0.90 | 0.01 | 0.07 |
| 18 | 18:13146999 | 6.61 | 0.90 | 0.01 | 0.07 |
| 18 | 18:13147063 | 6.61 | 0.90 | 0.01 | 0.07 |
| 18 | 18:13147747 | 6.61 | 0.90 | 0.01 | 0.07 |
| 18 | 18:13149017 | 6.61 | 0.90 | 0.01 | 0.07 |
| 18 | 18:13149305 | 6.61 | 0.90 | 0.01 | 0.07 |
| 18 | 18:13151402 | 6.61 | 0.86 | 0.01 | 0.07 |
| 18 | 18:13151967 | 6.61 | 0.86 | 0.01 | 0.07 |
| 18 | 18:13152843 | 6.61 | 0.86 | 0.01 | 0.07 |
| 18 | 18:13155943 | 6.61 | 0.83 | 0.01 | 0.07 |
| 18 | 18:13175633 | 6.93 | 0.90 | 0.01 | 0.07 |
| 18 | 18:13175950 | 6.93 | 0.89 | 0.01 | 0.07 |
| 18 | 18:13391752 | 9.84 | 0.83 | 0.02 | 0.11 |
| 18 | 18:13391841 | 9.84 | 0.83 | 0.02 | 0.11 |
| 18 | 18:13393733 | 9.84 | 0.85 | 0.01 | 0.11 |
| 18 | 18:13403337 | 10.57 | 0.83 | 0.02 | 0.11 |
| 18 | 18:13403968 | 9.37 | 0.87 | 0.01 | 0.10 |
| 18 | 18:13405460 | 9.37 | 0.87 | 0.01 | 0.10 |
| 18 | 18:13408106 | 10.57 | 0.85 | 0.02 | 0.11 |
| 18 | 18:13409996 | 10.57 | 0.84 | 0.01 | 0.10 |
| 18 | 18:13450556 | 10.77 | 0.80 | 0.02 | 0.11 |
| 18 | 18:13453819 | 10.77 | 0.82 | 0.01 | 0.10 |
| 18 | 18:13454607 | 10.77 | 0.95 | 0.02 | 0.11 |
| 18 | 18:13839520 | 10.08 | 0.80 | 0.01 | 0.09 |
|  |  |  |  |  |  |
| Chromosome | Name of variant | -Log_10_  (Pvalue) | AR2^§^ | $\sigma_{marker}^{2}$^¢^ | $\frac{\sigma_{marker}^{2}}{\sigma_{p}^{2}}$^*^ |
| 18 | 18:13840950 | 6.02 | 0.86 | 0.01 | 0.07 |
| 18 | 18:13934348 | 10.08 | 0.84 | 0.02 | 0.11 |
| 18 | 18:13934429 | 10.08 | 0.82 | 0.01 | 0.10 |
| 18 | 18:13934546 | 10.08 | 0.87 | 0.02 | 0.11 |
| 18 | 18:13934657 | 10.08 | 0.93 | 0.01 | 0.10 |
| 18 | 18:13934670 | 10.08 | 0.95 | 0.02 | 0.11 |
| 18 | 18:13934856 | 10.08 | 1.00 | 0.01 | 0.11 |
| 18 | 18:13934858 | 10.08 | 0.97 | 0.02 | 0.11 |
| 18 | 18:13934872 | 10.08 | 0.97 | 0.01 | 0.11 |
| 18 | 18:13934903 | 10.08 | 0.94 | 0.02 | 0.11 |
| 18 | 18:13934926 | 10.08 | 0.95 | 0.02 | 0.11 |
| 18 | 18:13935065 | 10.08 | 0.95 | 0.02 | 0.11 |
| 18 | 18:13935102 | 10.08 | 0.94 | 0.02 | 0.11 |
| 18 | 18:13935106 | 10.08 | 0.94 | 0.02 | 0.11 |
| 18 | 18:13935269 | 10.08 | 0.92 | 0.01 | 0.10 |
| 18 | 18:13935300 | 10.08 | 0.93 | 0.01 | 0.11 |
| 18 | 18:13935356 | 10.08 | 0.84 | 0.01 | 0.11 |
| 18 | 18:13935590 | 10.08 | 0.90 | 0.02 | 0.11 |
| 18 | 18:13938211 | 10.08 | 0.86 | 0.02 | 0.11 |
| 18 | 18:13938277 | 10.08 | 0.90 | 0.01 | 0.10 |
| 18 | 18:13938283 | 10.08 | 0.90 | 0.01 | 0.10 |
| 18 | 18:13938291 | 10.08 | 0.91 | 0.01 | 0.10 |
| 18 | 18:13938461 | 10.08 | 0.85 | 0.01 | 0.09 |
| 18 | 18:13938602 | 10.08 | 0.99 | 0.02 | 0.11 |
| 18 | 18:13938614 | 10.08 | 0.99 | 0.02 | 0.11 |
| 18 | 18:13938680 | 10.08 | 0.99 | 0.02 | 0.11 |
| 18 | 18:13938708 | 10.08 | 0.95 | 0.01 | 0.10 |
| 18 | 18:13938871 | 10.08 | 0.99 | 0.02 | 0.11 |
| 18 | 18:13938963 | 10.08 | 1.00 | 0.02 | 0.11 |
| 18 | 18:13939032 | 10.08 | 0.95 | 0.01 | 0.10 |
| 18 | 18:13939085 | 10.08 | 0.91 | 0.01 | 0.10 |
| 18 | 18:13939109 | 10.08 | 0.96 | 0.01 | 0.10 |
| 18 | 18:13939170 | 10.08 | 1.00 | 0.01 | 0.11 |
| 18 | 18:13939213 | 10.08 | 0.96 | 0.01 | 0.10 |
| 18 | 18:13939414 | 10.08 | 1.00 | 0.01 | 0.11 |
| 18 | 18:13939492 | 10.08 | 0.96 | 0.01 | 0.10 |
| 18 | 18:13939541 | 10.08 | 0.89 | 0.01 | 0.10 |
| 18 | 18:13940296 | 10.08 | 0.96 | 0.02 | 0.11 |
| 18 | 18:13941584 | 10.08 | 0.89 | 0.01 | 0.10 |
| 18 | 18:13941841 | 10.08 | 0.91 | 0.01 | 0.11 |
| Chromosome | Name of variant | -Log_10_  (Pvalue) | AR2^§^ | $\sigma_{marker}^{2}$^¢^ | $\frac{\sigma_{marker}^{2}}{\sigma_{p}^{2}}$^*^ |
| 18 | 18:13942012 | 10.08 | 0.90 | 0.01 | 0.10 |
| 18 | 18:13943200 | 10.08 | 0.93 | 0.02 | 0.11 |
| 18 | 18:13943440 | 10.08 | 1.00 | 0.01 | 0.11 |
| 18 | 18:13944067 | 10.08 | 0.95 | 0.01 | 0.11 |
| 18 | 18:13944341 | 10.08 | 0.95 | 0.01 | 0.11 |
| 18 | 18:13944359 | 10.08 | 0.95 | 0.01 | 0.11 |
| 18 | 18:13944405 | 10.08 | 0.95 | 0.01 | 0.11 |
| 18 | 18:13944426 | 10.08 | 0.94 | 0.01 | 0.11 |
| 18 | 18:13944487 | 10.08 | 0.94 | 0.01 | 0.11 |
| 18 | 18:13944678 | 10.08 | 0.94 | 0.02 | 0.11 |
| 18 | 18:13944759 | 10.08 | 0.93 | 0.01 | 0.11 |
| 18 | 18:13944979 | 10.08 | 0.97 | 0.02 | 0.11 |
| 18 | 18:13945037 | 10.08 | 0.92 | 0.01 | 0.11 |
| 18 | 18:13945704 | 10.08 | 0.88 | 0.02 | 0.12 |
| 18 | 18:13945860 | 10.08 | 0.85 | 0.02 | 0.11 |
| 18 | 18:13945962 | 10.08 | 0.86 | 0.02 | 0.12 |
| 18 | 18:13946128 | 10.08 | 0.88 | 0.02 | 0.11 |
| 18 | 18:13946143 | 10.08 | 0.87 | 0.02 | 0.11 |
| 18 | 18:13946439 | 10.08 | 0.85 | 0.01 | 0.10 |
| 18 | 18:13947029 | 10.08 | 0.84 | 0.02 | 0.12 |
| 18 | 18:13947133 | 10.08 | 0.84 | 0.02 | 0.12 |
| 18 | 18:13947135 | 10.08 | 0.84 | 0.02 | 0.12 |
| 18 | 18:13947191 | 10.08 | 0.84 | 0.02 | 0.12 |
| 18 | 18:13947229 | 10.08 | 0.83 | 0.02 | 0.12 |
| 18 | 18:13948757 | 10.08 | 0.86 | 0.02 | 0.11 |
| 18 | 18:13949676 | 10.08 | 0.83 | 0.02 | 0.11 |
| 18 | 18:13949754 | 10.08 | 0.85 | 0.02 | 0.11 |
| 18 | 18:13949853 | 10.08 | 0.85 | 0.02 | 0.12 |
| 18 | 18:13949912 | 10.08 | 0.85 | 0.02 | 0.12 |
| 18 | 18:13950098 | 10.08 | 0.85 | 0.02 | 0.12 |
| 18 | 18:13950100 | 10.08 | 0.85 | 0.02 | 0.12 |
| 18 | 18:13950384 | 10.08 | 0.85 | 0.02 | 0.12 |
| 18 | 18:13950481 | 10.08 | 0.85 | 0.02 | 0.12 |
| 18 | 18:13950512 | 10.08 | 0.85 | 0.02 | 0.12 |
| 18 | 18:13950714 | 10.08 | 0.86 | 0.02 | 0.12 |
| 18 | 18:13951417 | 10.08 | 0.90 | 0.02 | 0.11 |
| 18 | 18:13951454 | 10.08 | 0.90 | 0.02 | 0.11 |
| 18 | 18:13951584 | 10.08 | 0.83 | 0.02 | 0.12 |
| 18 | 18:13952060 | 10.08 | 0.90 | 0.02 | 0.11 |
|  |  |  |  |  |  |
| Chromosome | Name of variant | -Log_10_  (Pvalue) | AR2^§^ | $\sigma_{marker}^{2}$^¢^ | $\frac{\sigma_{marker}^{2}}{\sigma_{p}^{2}}$^*^ |
| 18 | 18:13952858 | 10.08 | 0.90 | 0.02 | 0.11 |
| 18 | 18:13953290 | 10.08 | 0.87 | 0.02 | 0.12 |
| 18 | 18:13953846 | 10.08 | 0.86 | 0.02 | 0.12 |
| 18 | 18:13953980 | 10.08 | 0.86 | 0.02 | 0.12 |
| 18 | 18:13954496 | 10.08 | 0.90 | 0.02 | 0.11 |
| 18 | 18:13955270 | 10.08 | 0.91 | 0.01 | 0.10 |
| 18 | 18:13955479 | 10.08 | 0.87 | 0.02 | 0.12 |
| 18 | 18:13956152 | 10.08 | 0.87 | 0.02 | 0.12 |
| 18 | 18:13956601 | 10.08 | 0.90 | 0.02 | 0.11 |
| 18 | 18:13956677 | 10.08 | 0.90 | 0.01 | 0.11 |
| 18 | 18:13956796 | 10.08 | 0.90 | 0.02 | 0.11 |
| 18 | 18:13956954 | 10.08 | 0.90 | 0.02 | 0.11 |
| 18 | 18:13957123 | 10.08 | 0.90 | 0.02 | 0.11 |
| 18 | 18:13957548 | 10.08 | 0.86 | 0.02 | 0.12 |
| 18 | 18:13957651 | 10.08 | 0.90 | 0.02 | 0.11 |
| 18 | 18:13957672 | 10.08 | 0.87 | 0.02 | 0.12 |
| 18 | 18:13958100 | 10.08 | 0.86 | 0.02 | 0.12 |
| 18 | 18:13958151 | 10.08 | 0.84 | 0.02 | 0.12 |
| 18 | 18:13958362 | 10.08 | 0.91 | 0.02 | 0.11 |
| 18 | 18:13958364 | 10.08 | 0.91 | 0.02 | 0.11 |
| 18 | 18:13958689 | 10.08 | 0.92 | 0.02 | 0.11 |
| 18 | 18:13958726 | 10.08 | 0.92 | 0.02 | 0.11 |
| 18 | 18:13959429 | 10.08 | 0.92 | 0.02 | 0.11 |
| 18 | 18:13959552 | 10.08 | 0.92 | 0.02 | 0.11 |
| 18 | 18:13959862 | 10.08 | 0.92 | 0.02 | 0.11 |
| 18 | 18:13959864 | 10.08 | 0.92 | 0.02 | 0.11 |
| 18 | 18:13960117 | 10.08 | 0.93 | 0.02 | 0.11 |
| 18 | 18:13960334 | 10.08 | 0.94 | 0.02 | 0.11 |
| 18 | 18:13960525 | 10.08 | 1.00 | 0.01 | 0.11 |
| 18 | 18:13961532 | 10.08 | 0.91 | 0.01 | 0.11 |
| 18 | 18:13962136 | 10.08 | 0.97 | 0.02 | 0.11 |
| 18 | 18:13962696 | 10.08 | 0.96 | 0.02 | 0.11 |
| 18 | 18:13962940 | 10.08 | 0.96 | 0.02 | 0.11 |
| 18 | 18:13962990 | 10.08 | 0.93 | 0.01 | 0.11 |
| 18 | 18:13963215 | 10.08 | 0.96 | 0.02 | 0.11 |
| 18 | 18:13964657 | 10.08 | 0.88 | 0.01 | 0.11 |
| 18 | 18:13965595 | 10.08 | 0.93 | 0.02 | 0.11 |
| 18 | 18:13967836 | 10.08 | 0.94 | 0.02 | 0.11 |
| 18 | 18:13967910 | 10.08 | 1.00 | 0.01 | 0.11 |
| 18 | 18:13968028 | 10.08 | 0.93 | 0.02 | 0.11 |
|  |  |  |  |  |  |
| Chromosome | Name of variant | -Log_10_  (Pvalue) | AR2^§^ | $\sigma_{marker}^{2}$^¢^ | $\frac{\sigma_{marker}^{2}}{\sigma_{p}^{2}}$^*^ |
| 18 | 18:13970606 | 10.08 | 0.80 | 0.02 | 0.12 |
| 18 | 18:13970771 | 10.08 | 0.80 | 0.01 | 0.10 |
| 18 | 18:13971413 | 10.08 | 0.86 | 0.02 | 0.11 |
| 18 | 18:15017933 | 10.31 | 0.99 | 0.01 | 0.11 |
| 18 | 18:15017982 | 9.24 | 1.00 | 0.01 | 0.10 |
| 18 | 18:15018610 | 9.24 | 0.99 | 0.01 | 0.10 |
| 18 | 18:15019735 | 10.31 | 0.99 | 0.01 | 0.11 |
| 18 | 18:15024959 | 10.31 | 0.83 | 0.01 | 0.10 |
| 18 | 18:15029101 | 14.12 | 0.88 | 0.02 | 0.14 |
| 18 | 18:15032047 | 14.12 | 0.88 | 0.02 | 0.14 |
| 18 | 18:15038074 | 7.05 | 0.85 | 0.01 | 0.07 |
| 18 | 18:15046094 | 7.05 | 0.89 | 0.01 | 0.07 |
| 18 | 18:15047436 | 6.46 | 0.99 | 0.01 | 0.07 |
| 18 | 18:15047675 | 6.46 | 1.00 | 0.01 | 0.07 |
| 18 | 18:15047877 | 6.46 | 0.99 | 0.01 | 0.07 |
| 18 | 18:15047927 | 6.46 | 0.99 | 0.01 | 0.07 |
| 18 | 18:15049190 | 7.05 | 0.84 | 0.01 | 0.08 |
| 18 | 18:15051124 | 7.05 | 0.86 | 0.01 | 0.08 |
| 18 | 18:15055682 | 7.05 | 0.84 | 0.01 | 0.08 |
| 18 | 18:15056537 | 7.05 | 0.87 | 0.01 | 0.08 |
| 18 | 18:15064047 | 6.68 | 0.89 | 0.01 | 0.08 |
| 18 | 18:15081850 | 6.68 | 0.96 | 0.01 | 0.08 |
| 18 | 18:15083765 | 6.68 | 0.96 | 0.01 | 0.08 |
| ^†^NC milk as binary trait where 0= coagulating and 1=non-coagulating | | | | | |
| ^§^AR2 =accuracy of imputation obtained from Beagle 4.0 | | | | | |
| ^¢^$\sigma_{marker}^{2}$ = marker’s variance, computed for each marker as 2 times major allele frequency times minor allele  frequency times allele substitution effect | | | | | |
| ^*^${\sigma_{marker}^{2}}/{\sigma_{p}^{2}}$ = phenotypic variance explained by a marker | | | | | |
